# Health and labor force participation among older workers in Switzerland: a growth curve analysis

**DOI:** 10.1007/s10433-022-00716-z

**Published:** 2022-08-12

**Authors:** Sonja Feer, Oliver Lipps, Julia Dratva, Isabel Baumann

**Affiliations:** 1 Institute of Public Health, Zurich University of Applied Sciences, Katharina-Sulzer-Platz 9, 8401 Winterthur, Switzerland; 2 Swiss Centre of Expertise in the Social Sciences (FORS), Lausanne, Switzerland; 3 Medical Faculty, University of Basel, Basel, Switzerland; 4 Institute of Sociology, University of Bern, Bern, Switzerland; 5 Centre Interfacultaire de Gérontologie Et d’Etudes de Vulnerabilité and NCCR LIVES, University of Geneva, Geneva, Switzerland

**Keywords:** Self-rated health, Working hours, Multilevel analysis, Occupational groups, Older workers

## Abstract

This study investigated how individual trajectories of self-rated health (SRH) and working hours among older workers in Switzerland are interrelated and how this relationship varies based on occupation. We used data from the Swiss Household Panel to analyze the long-term trajectories of older workers measured in terms of working hours and SRH. The sample included more than 4000 workers aged 50 to 65(men)/64(women). We ran a bivariate response multilevel model for growth that allowed the examination of between- and within-individual changes over time. On a between-individual level, we found that the upper non-manual workers were the most heterogeneous occupational group in terms of working hours and the lower non-manual workers were the most heterogeneous occupational group in terms of health. Within all occupational groups, we found a significant relationship between the level of working hours and level of SRH. The individual-level statistics showed consistently strongest effects for manual workers. This result confirms our hypothesis that labor force participation in individuals of the manual occupational group is more sensitive to their health status. Our findings contribute to the debate regarding the importance of older workers’ health in the context of the extension of working life.

## Introduction

Increasing financial pressure on old-age pension systems has caused many countries belonging to the Organisation for Economic Co-operation and Development (OECD) to raise the eligible age for old-age pension benefits (OECD 2019). In Switzerland, where the eligible age has not been raised for the last 15 years, policy measures to ensure the financial sustainability of old-age pension systems are currently under debate. Most measures are aiming to extend the labor force participation of older workers, and subsequently, their financial contributions to old-age pensions (Bello and Galasso 2021; Bütler and Engler 2008). For instance, the women’s age of eligibility of 64 may be adjusted the men’s age of 65, and partial pensions may be introduced.

While these measures are likely to increase the financial sustainability of the pension system, whether older workers’ health allows for an extension of working life remains an open question. Previous research indicated that older workers with poor health are more likely to exit the labor force prematurely (Johnson 2018; Jokela et al. 2010; Mein et al. 2000; Schirle 2010), and those with good health are more likely to extend their working life after reaching the statutory retirement age (Scharn et al. 2017; von Bonsdorff et al. 2010), a phenomenon referred to as health selection (Heggebø 2015). A broader definition of health selection encompasses both entry to and exit from the labor force (Heggebø 2015). In this study, we use the term in an even broader sense by including reductions or increases in working hours. This approach allows us to study the relationship between self-rated health (SRH) and fine-grained changes in labor force participation.

Although a large body of literature on health selection exists, few studies have investigated the heterogeneous effects of health selection on labor force exit, that is, whether some specific subgroups are more likely than others to leave the labor force prematurely due to poor health. Among the few exceptions is a longitudinal study based on Swiss data that examined health-related early retirement (Dorn and Sousa-Poza 2004). The study found differences between occupational groups. In particular, it showed that craftsmen, machine operators, assemblers, and workers in elementary occupations (groups 7–9 according to the International Standard Classification of Occupations [ISCO]) were significantly more likely than the reference group of clerks to retire early for health reasons (Dorn and Sousa-Poza 2004). A study from Finland reported a lower working life expectancy for older manual workers due to ill health-related reasons (Schram et al. 2021). A qualitative study from the Netherlands concluded that workers in physically demanding blue-collar occupations were more likely to retire early due to health reasons (Reeuwijk et al. 2013). The interviews revealed that blue-collar workers already sensed the deterioration of their body around age 40 and thus quit the labor force early. However, although these studies examined differences between occupational groups in terms of retirement behavior, they did not consider the potential variation within each occupational group and over time.

Another study analyzed heterogeneous effects in health-related labor force exit, although not specifically in older workers (Heggebø 2015). Comparing three Scandinavian countries, the author found no differences between genders and educational groups but significant differences between age groups. Individuals below the age of 30 were more likely to exit the labor force due to poor health than older workers and often ended up unemployed. Another study from the United Kingdom examined how health-related reasons for retirement are associated with the long-term health outcomes of retirees and found that retirement due to ill health was related to poorer mental health (Jokela et al. 2010). Yet another series of studies examined heterogeneity retirement timing, although without a specific focus on health (Madero-Cabib et al. 2016; Madero-Cabib and Kaeser 2016). One study showed that the timing of retirement was associated with older workers’ pension savings, in particular with occupational pension savings (Madero-Cabib and Kaeser 2016). People with occupational pensions were more likely than those without occupational pensions to retire early or on time (i.e., at the statutory retirement age). Another study showed that people with interrupted occupational careers and part-time employment were less likely to retire early and more likely to retire late (Madero-Cabib et al. 2016). These results suggest that workers’ retirement savings importantly influence the timing of retirement. Workers who are in a less advantageous financial situation may not have the same scope of action in retirement behavior if they experience health problems.

This implication is relevant in the context of the Swiss labor market, which is characterized by substantial gender differences in part-time employment (Madero-Cabib 2016). In 2000, 54% of women and 10% of men of working age worked part-time (OECD 2021). In 2020, these percentages increased to 59% of women and 18% of men. Among older workers aged 55–65, the proportion of the population engaged in part-time labor was 67% (women) and 11% (men) in 2000, which increased to 69% (women) and 18% (men) in 2020. Part-time employment in Switzerland is associated with lower levels of pension savings in the occupational pension scheme (Madero-Cabib 2016). On the one hand, this is due to lower levels of annual income, which lead to lower annual contributions to occupational pension plans, and on the other hand, to an income threshold below which there are no contributions to occupational pension plans.

This study builds on previous evidence by investigating how *within*-individual changes in SRH and working hours differ *between* individuals aged 50 to 64 or 65 (i.e., statutory retirement age for women and men, respectively) for three different occupational groups. We used a bivariate response multilevel model for growth and controlled for age, education, and gender. The advantage of the model for growth over other longitudinal methods is that *within*- and *between*-individual changes can be assessed at the same time and that two outcome measures (i.e., SRH and working hours) are modeled simultaneously (Curran et al. 2010).

Based on previous studies that showed that older workers in physically more strenuous occupations are more likely to experience health-related early labor force exit, we hypothesize that within-individual associations between SRH and working hours between individuals in manual occupations vary more strongly than in other occupations, and this variation increases the closer individuals get to the statutory retirement age. More briefly, we expect that SRH and working hours are more strongly related among manual workers. The rationale of this hypothesis is that workers in manual occupations are not only more likely to experience poor health than workers in other occupational groups but that their labor force participation is also more strongly, and over time, increasingly affected by their potentially poor health.

## Methods

### Data

We used data from waves 1999–2020 of the Swiss Household Panel (SHP; see Tillmann et al. 2016) for details). The SHP is an annual, centrally conducted nationwide computer-assisted telephone interview (CATI) panel survey that uses a stratified random sample of the Swiss residential population. Initially conducted in 1999 with more than 5000 households, the SHP added two refreshment samples: one in 2004 with more than 2500 households and one in 2013 with about 4000 households. In their respective first waves, the 1999 original sample reached a household-level response rate of 64%, the 2004 refreshment sample attained a rate of 65%, and the 2013 refreshment sample had a rate of 60% (Response Rate 1 [RR1]; AAPOR 2016). Each year, the reference person of each household was asked to complete the household grid questionnaire, and then the household questionnaire was followed by all household members aged 14 or over being interviewed using an individual questionnaire. The SHP contains a wide range of questions about health, well-being, attitudes, social networks and economics. Our sample consists of all respondents aged 50 and over until statutory retirement age in Switzerland (65 years for men and 64 years for women) with nonzero working hours at baseline. We imputed the missing occupations for respondents who were out of the labor force with the last occupation or with the closest given valid previous or next occupation if unemployed or temporarily not working. After deleting missing cases on the other variables list wise (see measures below), the total number of individuals was 4,345, and the number of observations was 14,625. The descriptive statistics are presented in Table 1.

**Table 1:** Sociodemographic data on the Swiss Household panel sample

Age: mean (SD)^a^	57.7 (4.0)
Weekly working hours: mean (SD)^a^	35.5 (15.8)
*Gender*	
Female	43.9%
Male	56.1%
*Education*	
Tertiary	32.8%
Upper secondary	46.5%
Below upper secondary	20.8%
*Occupation, according to ISCO*^b^ *classification*	
Upper non-manual (ISCO 1–2)	32.8%
Lower non-manual (ISCO 3–5)	46.5%
Manual (ISCO 6–9)	20.8%
*Self-rated health*	
Very well (4)	22.7%
Well (3)	62.7%
So-so (average) (2)	12.9%
Not very well (1)	1.5%
Not well at all (0)	0.2%

Total of 14,625 observations from 4,345 persons from 1999 to 2020

^a^*SD—*standard deviation

^b^*ISCO*—International Standard Classification of Occupations

### Measures

Our two dependent variables are working hours and SRH. Working hours were measured on a scale from 0 to 99 and were assessed in the SHP with the question “How many hours do you usually work each week for your main job?” (Variable P$$W77).

Health status was measured in terms of SRH. SRH was assessed using the question “How do you feel right now?” Possible answers to this question were “very well” (recoded to 4), “well” (3), “so-so (average)” (2), “not very well” (1), and “not well at all” (0). SRH has been shown to have satisfactory reliability and validity (Cox et al. 2009; DeSalvo et al. 2006; Idler et al. 2000) and has been used in numerous studies to assess health status (Remund et al. 2019).

Our independent variable was occupation. Occupation was measured in terms of ISCO 2008 (ISCO-08). Occupations were categorized at the 1-digit level of the ISCO-08 into three groups—manual workers (groups 6–9), lower non-manual workers (groups 3–5), and upper non-manual workers (groups 1–2). This categorization corresponds to a collapsed version of the categorization applied by Hessels et al. (2018) or Anna et al. (2018). If individuals changed occupation across waves, we used the most often reported occupation as a basis for the categorization into groups.

### Analysis

The growing availability of panel data is increasingly allowing researchers to apply sophisticated longitudinal methods. We used a bivariate response multilevel model for growth that allowed us to simultaneously analyze *within*- and *between*-individual patterns of change (Singer and Willett 2003). This approach allowed us to assess the level of heterogeneity of the trajectories of the last 15 years before reaching statutory retirement age and how this level of heterogeneity changed over time (Cullati 2015). Furthermore, models for growth exceled in examining the multivariate growth processes (i.e., allowing simultaneous analysis of multiple dependent variables), non-normally distributed repeated measures, or nonlinear trajectories, which made them particularly appropriate for the analysis of our data (Curran et al. 2010). Simultaneous modeling of SRH and working hours yielded additional estimates of interesting associations between both variables and may also help to address the problem of health selection (Lindeboom and Kerkhofs 2009).

Our analysis provided estimates for both fixed and random effects. Fixed effects are estimated coefficients of covariates, while random effects denote the variances and covariances of residuals on different levels (Singer and Willett 2003). In models of growth, the fixed effects represent the mean of the trajectory of individuals with specific characteristics (Curran et al. 2010). The random effects represent the between-person variances at the individual levels (i.e., intercepts) and slopes. Smaller variances imply that the trajectories are more similar across the sample.

We included working hours and SRH as dependent variables and occupation as the independent variable. We controlled for age, age squared, gender, education (measured as a linear variable with 11 categories), and interaction effects between occupation and age and between occupation and age squared. We controlled for age squared to model the effects of age more accurately because we assumed a nonlinear relationship with the dependent variable.

## Results

**Fig. 1:**
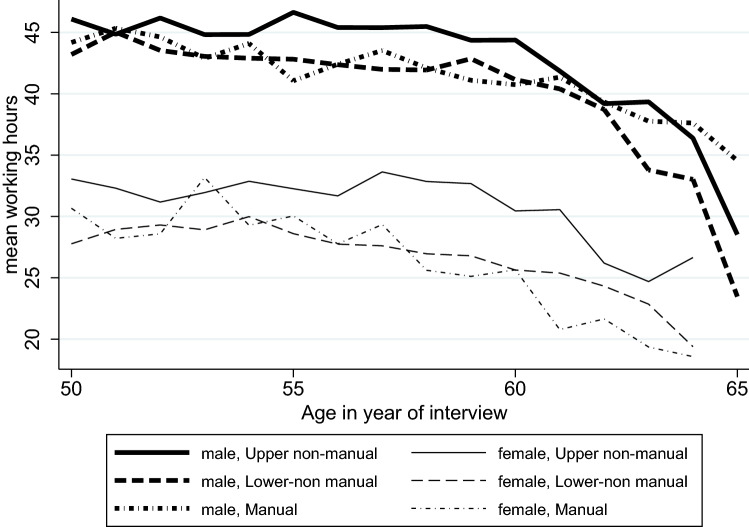
Working hours by age and gender; Swiss Household Panel (1999–2020)

Figure 1 illustrates the distribution of the dependent variable of working hours by age and gender. Women worked on average fewer hours than men, and their decrease in working hours in the years before the statutory retirement age was less drastic than that among men. The differences in working hours between occupations were greater among women than men, particularly in the last few years before retirement. Between both genders, upper non-manual workers generally worked more hours than lower non-manual and manual workers. However, this pattern in men changed during the last years before reaching the statutory retirement age, where manual workers were less likely than non-manual workers to reduce their working hours. Among women, there was slightly more variation between occupational groups than that among men, in particular at the end of the observation period.

**Fig. 2:**
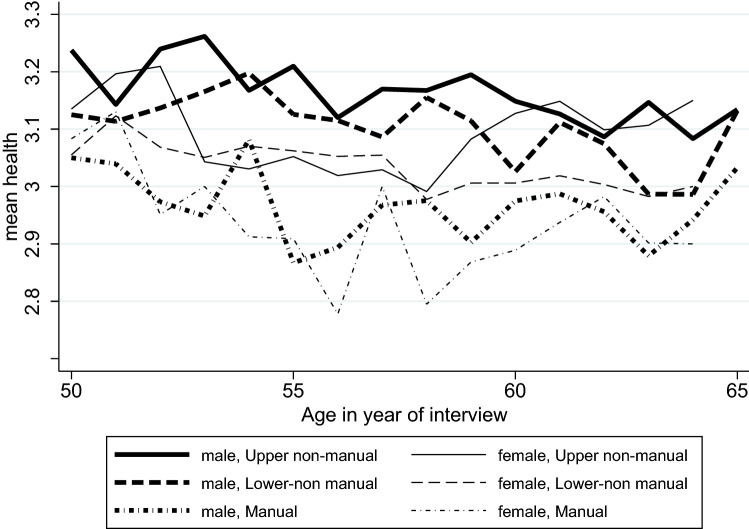
Self-rated health by age and gender; Swiss Household Panel (1999–2020)

Figure 2 illustrates the distribution of the dependent variable SRH by age and gender. In contrast to the case of working hours (Fig. 1), there was no clear difference in SRH between women and men (Fig. 2). However, manual workers showed comparably low levels of SRH, and male upper non-manual workers showed comparably high levels of SRH.

### Fixed effects

**Fig. 3:**
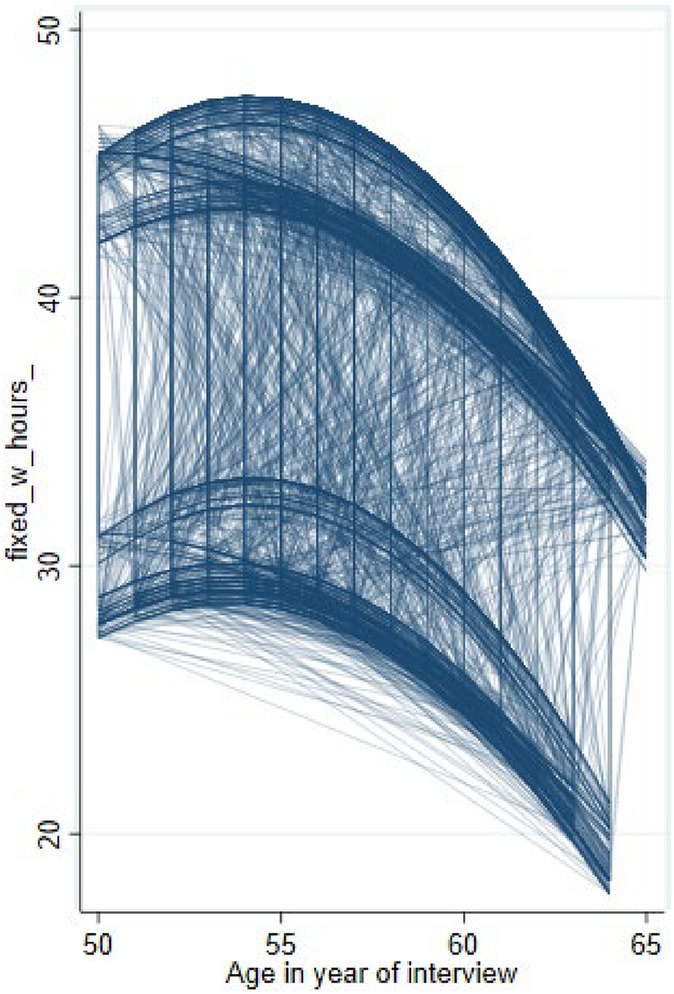
Individual trajectories of fixed effects results from the association between age and working hours; SHP (1999–2020)

The results of the fixed effects analyses of the multilevel model for growth are listed in Table 2. In the analysis of the *trajectories of working hours*, we found that men (14.223; *p* < 0.001) and people with higher levels of education (0.165; *p* < 0.001) worked more hours. Manual workers (30.567; *p* < 0.001) worked more hours than upper non-manual workers (29.419; *p* < 0.001), who in turn worked more hours than lower non-manual workers (27.166; *p* < 0.001). Age was positively associated with working hours in the non-manual working groups; this association was the highest among upper non-manual workers. The association between age and working hours varied between individuals, and the trajectories were generally inverted U-shaped (Fig. 3 in the appendix). All occupational groups showed a negative interaction with age squared. This finding implies a concave growing curve for working hours over time. The multivariate analysis confirmed the descriptive result from Fig. 1: Men worked more hours than women do. However, with respect to the occupational groups, the multivariate analysis showed reversed results as compared to the descriptive results: Manual workers worked more hours than the other occupational groups do.

**Table 2:** Fixed effects results from the multilevel model for growth

	Working hours		Self-rated health	
	Coef	SE	Coef	SE
Male	14.223***	0.393	0.070***	0.0018
Education	0.165***	0.072	0.016***	0.003
Occupation				
Manual (ISCO 6–9)	30.567***	0.992	2.946***	0.0046
Lower non-manual (ISCO 3–5)	27.166***	0.656	3.063***	0.033
Upper non-manual (ISCO 1–2)	29.419***	0.919	3.106***	0.043
Manual × age	− 0.040	0.224	− 0.024**	0.011
Lower non-manual × age	0.713***	0.130	− 0.016	0.008
Upper non-manual × age	1.055***	0.166	− 0.022**	0.008
Manual × age squared	− 0.053***	0.014	0.001	0.001
Lower non-manual × age squared	− 0.100***	0.008	0.000	0.000
Upper manual × age squared	− 0.123***	0.010	0.000	0.001

Data: Swiss Household Panel (1999–2020). *N* = 14,625 observations (4345 individuals)

*p < 0.05; **p < 0.01; and ***p < 0.001

**Fig. 4:**
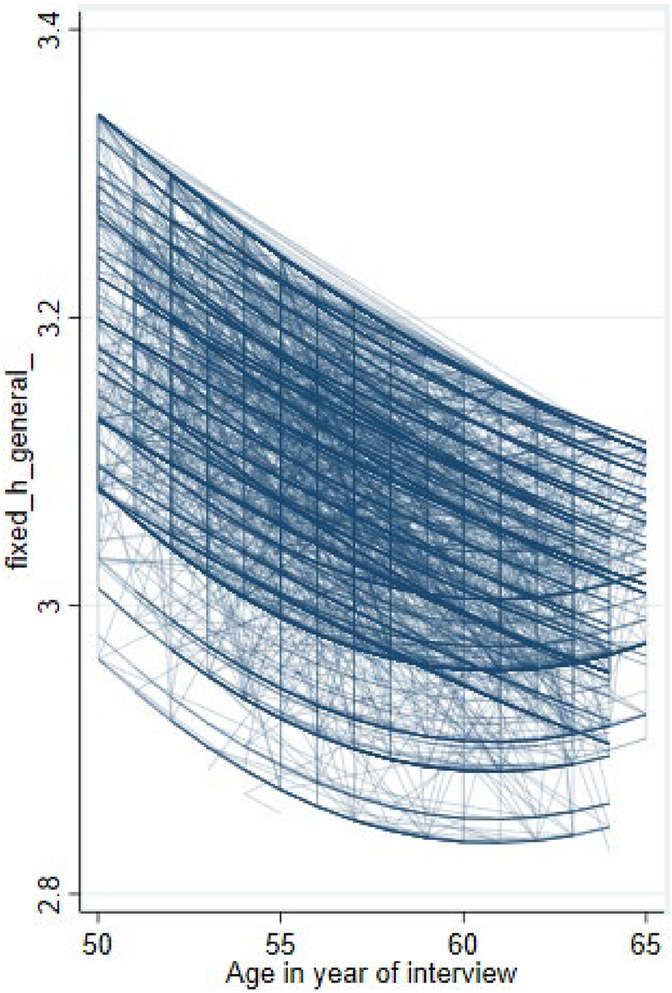
Individual trajectories of fixed effects results from the association between age and health; SHP (1999–2020)

In the analysis of the *trajectories of SRH*, we found that men (0.070; *p* < 0.001) and people with higher levels of education (0.016; *p* < 0.001) reported better health. Upper non-manual workers (3.106; *p* < 0.001) reported better health than lower non-manual workers (3.063; *p* < 0.001), who in turn reported slightly better health than manual workers (2.946; *p* < 0.001). Age was negatively associated with health in all occupational groups, with the most negative association being among manual workers compared to the other occupational groups. The association between age and health varied between individuals and the trajectories showed a general, slightly convex curvature (Fig. 4). The multivariate analysis confirmed the results from Fig. 2 that manual workers reported the lowest levels of health, but this analysis yielded the additional result that men reported better health than women.

### Random effects—between-individual level

**Fig. 5:**
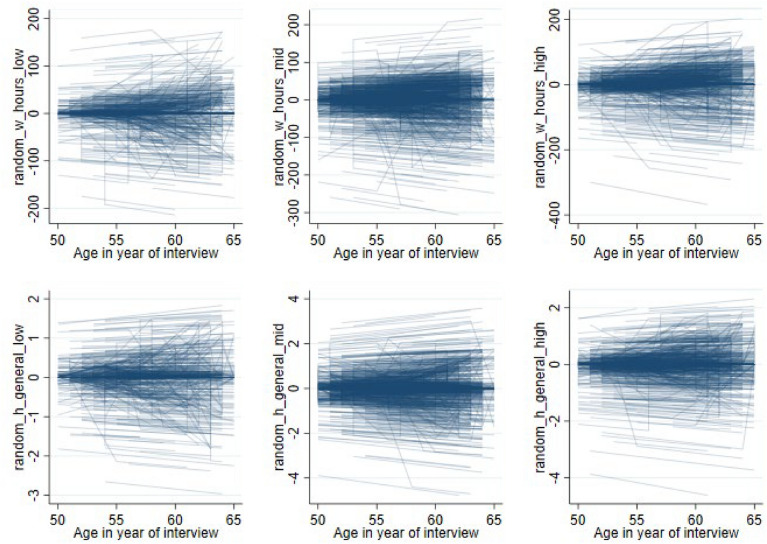
Individual trajectories of random effects results from the multilevel model for growth; SHP (1999–2020)

Table 3 shows the results of the random effects of the multilevel model for growth (Fig. 5 in the appendix illustrates the results of the individual trajectories of the random effects). First, we report the between-individual-level results. Specifically, we estimated the between-individual variances and covariances in SRH and working hours across different occupational groups. The variance in the level of a measure indicated its variance at baseline; the variance of the slope of a measure indicated its variance of change over time.

**Table 3:** Random effects results from the multilevel model for growth

	Manual (ISCO 6–9)		Lower non-manual (ISCO 3–5)		Upper non-manual (ISCO 1–2)	
	Est	SE	Est	SE	Est	SE
*Between individuals (level 2: idpers)*						
Working hours (W)						
var (level (W))	159.636*	16.537	151.092*	9.188	167.721*	12.906
var (slope (W))	1.746*	0.242	2.036*	0.153	2.290*	0.204
cov (level (W), slope (W))	− 7.965*	1.819	− 11.326*	1.101	− 13.668*	1.529
Self-rated health (H)						
var (level (H))	0.199*	0.030	0.246*	0.022	0.227*	0.024
var (slope (H))	0.001*	0.000	0.001*	0.000	0.001*	0.000
cov (level (H), slope (H))	− 0.004	0.003	− 0.011*	0.002	− 0.007*	0.002
Working hours and self-rated health combined (W, H)						
cov (level (W), level (H))	1.020*	0.503	− 0.695*	0.317	1.284*	0.398
cov (slope (W), level (H))	0.006	0.067	0.158*	0.045	− 0.139*	0.055
cov (level (W), slope (H))	− 0.094	0.058	0.064*	0.039	− 0.108	0.044
cov (slope (W), slope (H))	0.008	0.006	− 0.008	0.005	0.017*	0.005
*Within individuals (level 1: age)*						
Working hours (W)						
var (level(W))	71.887*	2.340	45.921*	1.009	56.651*	1.461
Self-rated health (H)						
var (level(H))	0.272*	0.009	0.247*	0.005	0.223*	0.006
Working hours and self-rated health combined (W, H)						
cov (level(W), level(H))	0.201*	0.100	0.022	0.052	0.039	0.064

Data: Swiss Household Panel (1999–2020). *N* = 14,625 observations (4345 individuals)

*p < 0.05

In the analysis of the level of *trajectories of working hours*, we found that the upper non-manual group was the most heterogeneous of all occupational groups at baseline. With respect to the slope of *trajectories of working hours*, the upper non-manual group showed the strongest change in variance across all occupational groups. The covariance between the level and slope of the *trajectories of working hours* was negative among all occupational groups. This implies that there was convergence in working hours within all occupational groups. This convergence was largest for upper non-manual workers.

Analyzing the variance of the level of *trajectories of SRH*, we found that the lower non-manual group was the most heterogeneous of all occupational groups at baseline. The slopes of *trajectories of SRH* indicated that all occupational groups remained homogeneous to the same degree over time. The covariance between the levels and slopes of *trajectories of SRH* was negative among all occupational groups (although not statistically significant among manual workers). The negative covariance implied that there was a convergence in SRH within the occupational group. This convergence was largest for lower non-manual workers.

We now turn to the results of the analysis of the *trajectories of working hours and SRH combined.* First, the covariance between the level of working hours and the level of SRH was negative for lower non-manual workers. This implies that the greater the number of working hours at baseline, the lower the SRH at baseline (or vice versa). This covariance was positive for manual and upper non-manual workers, with a stronger effect among upper non-manual workers. Second, the covariance between the slope of working hours and the level of SRH was negative for upper non-manual workers. This implies that higher levels of SRH at baseline were related to more negative changes in working hours. This covariance was positive for lower non-manual workers and insignificant for manual workers. Third, the covariance between the level of working hours and the slope of SRH was positive for lower non-manual workers. This implies that higher levels of working hours were related to more positive changes in SRH. This covariance was insignificant for manual and upper non-manual workers. Fourth, the covariance between the slope of working hours and the slope of SRH was positive for upper non-manual workers. This implies that more positive changes in working hours were related to more positive changes in SRH. This covariance was insignificant for manual and lower non-manual workers.

### Random effects—within-individual level

Second, we analyzed the within-individual effect. This effect represents the deviation from the individual trajectory of either SRH or working hours and how it is related to a deviation (in the same direction) of the other variable. The results of the analysis of the *trajectory of working hours* showed the highest variance within manual workers. *The trajectory of SRH again* showed the highest variance within manual workers. Finally, the *trajectory of working hours and SRH combined* was once again largest among manual workers and significant only in this occupational group. This implies that, among manual workers, we find the strongest sensitivity in one variable if the other is changed.

## Discussion

In summary, we showed that manual workers work the most hours and report the lowest levels of health among all occupational groups. The upper non-manual workers were the most heterogeneous occupational group in terms of working hours at baseline, but they experienced the largest convergence in working hours until they reached the statutory retirement age. However, lower non-manual workers were the most heterogeneous occupational group in terms of health at baseline and experienced the largest convergence until they reached the statutory retirement age. Within all occupational groups, we found a significant relationship between the level of working hours and the level of SRH at baseline. This relationship was positive among manual and upper non-manual workers and negative among lower non-manual workers. Finally, at the within-individual level, we consistently found the strongest effects for manual workers. Accordingly, among manual workers, the deviation from their individual health trajectory was strongest if there was a change in working hours, and the deviation from their individual working hours trajectory was strongest if there was a change in health. This implies that among manual workers, the trajectory of one measure (working hours or SRH) was more sensitive to changes in the other measure than among other occupational groups. Thus, we found evidence for our hypothesis that older workers’ health status and labor force participation are associated more strongly within individuals in manual occupations.

Our results extend the previous literature on health selection by focusing only on blue- and white-collar workers instead of white-collar workers and presenting the effects for three occupational groups (Jokela et al. 2010). A novel finding of our research is that large within-individual deviations from the individual SRH trajectory were associated with larger within-individual deviations from the individual working hour trajectories for manual workers, whereas no such effect could be observed for non-manual workers. This result may be explained by manual workers’ lack of transferrable skills (e.g., communication or social skills), which could be a limiting factor for their opportunities to change jobs or occupations in the case of a health problem. This interpretation is supported by Iversen and Cusack (2000), who argued that the skills of manufacturing workers transfer poorly to service jobs. A Swiss study on manufacturing workers laid off due to firm closure showed that those who worked in non-manual occupations—particularly those in upper non-manual occupations—were more likely to change occupations upon reemployment than manual workers (Baumann 2016). This effect was found to be more pronounced among older workers because they often have longer tenures, and therefore more firm-specific skills that reduce their skill transferability (Parrado et al. 2007).

Another potential explanation for our finding may be the main causes of health deterioration among lower occupational groups. Previous research showed that manual workers are particularly prone to suffering from musculoskeletal diseases, which are the leading cause of work disability and sickness absence, as well as productivity loss across European countries (Bevan 2015). For example, a Finnish study on sickness absence by occupation and reasons for sickness absence showed substantially higher risks of sickness absence for workers in manual occupations. Compared to other reasons for sickness absence, musculoskeletal diseases were found to contribute to significant differences in sickness absence between occupational groups (Leinonen et al. 2018). Among musculoskeletal diseases, back pain accounts for the highest rate of work absence (Bevan 2012). These findings indicate that a manual worker with back pain may more likely be restricted to continuing working as opposed to a non-manual employee with better working conditions. This is in line with research that showed that better working conditions may allow workers to continue work, despite a decrease in SRH (Dragano et al. 2016). Overall, older workers show higher number of sick days at work than younger workers (Gotz et al. 2018). Consequently, older workers in manual occupations are more likely to suffer from prolonged health-related work absences, which in turn may lead to premature health-related drop out from the labor market.

Although we found that the trajectory of either SRH or working hours was most sensitive to a change (in the same direction) within manual workers, we saw the highest number of working hours and the lowest level of SRH in this working group. A possible explanation for this may be that even though manual workers showed the lowest level of SRH and would therefore need a reduction in workload by reducing working hours, they might not be able to afford to reduce their working hours and must continue working full-time due to financial reasons. In contrast, upper non-manual workers might be able to afford a reduction in working hours according to their health needs throughout the last years before they reach the statutory retirement age. In fact, part-time employment parallels lower levels of annual income, which leads to lower levels of savings in the occupational pension scheme. Workers with no or low levels of savings, most often women, are less likely to retire before the statutory retirement age because they lack the savings to cover the time gap between early retirement and the beginning of their eligibility for payments from the public pension fund (Madero-Cabib 2016).

The finding that upper non-manual workers are the most heterogeneous occupational group in terms of working hours at baseline may be explained by the fact that the group of upper non-manual occupations encompasses managers [ISCO-08 group1, (e.g., finance managers)] and professionals [ISCO-08 group2, (e.g., architects)]. Workers in these occupations have been found to work long hours (Burke 2009; Feldman 2002; Statistisches Bundesamt 2010). However, not everybody chooses to work long hours. Workers from the upper non-manual occupational group may be more likely to afford to work part-time than workers from other occupational groups. This may lead to the largest variance in working hours among all of the occupational groups we examined. The strongest convergence among them is plausible given their initially highest level of heterogeneity, and many of them probably retire around the statutory retirement age.

Our results confirm that lower non-manual workers are the most heterogeneous occupational group in terms of health at baseline. Until they reach the statutory retirement age, they experience the largest convergence. This finding of heterogeneity at baseline may be explained by the fact that this was the largest of the occupational groups we examined, making up about 50% of our sample. Another explanation may be that it encompassed workers of different educational levels, which affects their health literacy and lifestyle (Sorensen et al. 2015). While technicians and associate professionals (ISCO-08 group 3, e.g., civil engineering technicians) may have tertiary education or at least upper secondary education, service and sales workers (ISCO-08 group 5, e.g., waiters) may have a lower level of education. With respect to our control variables, in addition to education, age and gender likewise influenced health-based labor market exit. This finding agrees with previous research that showed that age increases the probability of early retirement due to disability, and the female sex reduces the probability of early retirement (Dahl et al. 2003; Madero-Cabib and Kaeser 2016). Another indication of women’s lower probability of early retirement found in our data is their flatter drop in working hours in the years before the statutory retirement age. Another contrast between women and men is that the differences in the trajectories of working hours between the three occupational groups were larger among women than among men. This reflects the generally larger variances in women’s labor force participation compared to men’s (Finch 2014). In Switzerland, this variance is driven by the higher likelihood of women with children to work part-time, particularly those with a lower socioeconomic status (Wepfer et al. 2015).

Our study contributes to the literature on older workers’ health trajectories by using a sophisticated bivariate multilevel model for growth. In contrast to previous studies that focused only on the growth patterns of health trajectories (Lipps and Moreau-Gruet 2010) or examined whether individual health trajectories vary according to education and income (Della Bella et al. 2012), our analysis investigated the distinct trajectories of SRH and working hours of different occupational groups. While Cullati (2015) examined the role of labor market status in workers’ health trajectories, we included a more fine-grained measure of labor force participation among workers using working hours. Moreover, in contrast to other studies, we specifically examined workers above the age of 50 and thus provided novel evidence of the relationship between SRH and working hours in the years before retirement. Thus, we shed light on the mechanisms that lead workers to exit the labor force prematurely.

Our contribution should be viewed in light of the following limitations. First, our measure for health status, SRH, has been criticized in the past. Critics are concerned about the potential reporting bias in SRH. Poor health may be used as a justification for retirement. However, Mortelmans and Vannieuwenhuyze (2013), who used data for 15 countries from the European Community Household Panel, found no evidence of this assumption. Research has shown that SRH is a good indicator of objective health status (Wuorela et al. 2020) and that most indicators of medical and functional health are homogeneously associated with SRH (Bardage et al. 2005). Second, our method did not allow us to draw causal conclusions. Thus, we do not know whether change in one variable of our two outcome variables for SRH or working hours influenced changes in the other variable. However, the multilevel model for growth has the advantage of being able to model the two outcome measures of SRH and working hours simultaneously while assessing within- and between-individual change (Curran et al. 2010). Third, we did not differentiate between those who reduced their working hours while remaining in the labor force (i.e., reduction from a high activity level to a low activity level) and those who reduced their working hours while exiting the labor force (i.e., reduction from any activity level to zero working hours). The reason for our approach was that we were principally interested in studying the relationship between changes in working hours and health in older workers in general, and not specifically among those who exited the labor force or those who remained.

Yet, distinguishing between a complete labor force exit and a reduction in working hours may be a direction for future research. This approach may provide a better understanding of the mechanism leading from health problems to permanent labor force exits. Moreover, the inclusion of information about workers’ task and time flexibility may provide a more detailed picture of health-related triggers of early retirement. As Wang and Shultz (2009) argued, early retirement mechanisms may not be the same for all occupational groups, and early retirement decisions for blue-collar workers may be more dependent on the physical demands and negative health effects of their jobs.

## Conclusion and policy implications

Our study contributes to the ongoing academic and public debate regarding the labor force participation of older workers in the context of an aging workforce and an increasing retirement age. Our findings imply that if workers have to prolong their occupational careers, then supporting older workers with poor health and improving working conditions will be crucial. Given that manual workers experience the lowest levels of health, prevention of ill health may be particularly important for them. The current shortage of qualified workers in many sectors calls for measures that can maintain this specific group of older workers in the labor force. Furthermore, altering job roles may be an organizational measure to reduce job demands and improve working conditions in older workers. However, it is important that such measures be considered in conjunction with older workers’ health-related needs.

## Data Availability

This study used data collected by the Swiss Household Panel, which is based out of the Swiss Centre of Expertise in the Social Sciences (FORS).

## Appendix

See Figs. 3, 4 and 5.

## Code availability

Stata 16 SE was used for statistical analysis. The Stata program runmlwin (Leckie and Charlton 2013) was used to run the Multilevel Modelling Software MLwiN (Version 3.05).

## Declarations

### Data deposition information

This study used data from waves 1999–2020 of the Swiss Household Panel available at https://forsbase.unil.ch/project/study-public-overview/17411/0/.
